# Alternative forms for genomic clines

**DOI:** 10.1002/ece3.609

**Published:** 2013-05-23

**Authors:** Benjamin M Fitzpatrick

**Affiliations:** Department of Ecology and Evolutionary Biology, University of TennesseeKnoxville, Tennessee, 37996

**Keywords:** Admixture, hybrid zones, introgression, reproductive isolation, speciation

## Abstract

Understanding factors regulating hybrid fitness and gene exchange is a major research challenge for evolutionary biology. Genomic cline analysis has been used to evaluate alternative patterns of introgression, but only two models have been used widely and the approach has generally lacked a hypothesis testing framework for distinguishing effects of selection and drift. I propose two alternative cline models, implement multivariate outlier detection to identify markers associated with hybrid fitness, and simulate hybrid zone dynamics to evaluate the signatures of different modes of selection. Analysis of simulated data shows that previous approaches are prone to false positives (multinomial regression) or relatively insensitive to outlier loci affected by selection (Barton's concordance). The new, theory-based logit-logistic cline model is generally best at detecting loci affecting hybrid fitness. Although some generalizations can be made about different modes of selection, there is no one-to-one correspondence between pattern and process. These new methods will enhance our ability to extract important information about the genetics of reproductive isolation and hybrid fitness. However, much remains to be done to relate statistical patterns to particular evolutionary processes. The methods described here are implemented in a freely available package “HIest” for the R statistical software (CRAN; http://cran.r-project.org/).

## Introduction

Hybrid zones are natural experiments offering unique insights into evolution (Endler 1977; Hewitt 1988; Harrison 1990; Buerkle and Lexer 2008). In addition, hybridization is common in nature, and constitutes an important phenomenon impacting the evolution of diversity and novelty (e.g., Anderson 1948; Anderson and Stebbins 1954; Whitham 1989; Harrison 1993; Hewitt 2001; Arnold 2006; Arnold and Martin 2009). Hybrids and hybrid zones bring many new combinations of alleles together simultaneously, potentially leading to rapid evolution of multilocus novelties that would be difficult to evolve on a locus-by-locus basis (Rieseberg and Linder 1999; Rieseberg et al. 2003; Arnold 2006; Gompert et al. 2006; Mavarez et al. 2006; Fitzpatrick and Shaffer 2007a). Recombination in hybrid populations allows different loci to evolve differently depending on their linkage relationships and functional interactions with other loci.

An important question is to what extent different genes behave as members of a single “coadapted gene complex” (Dobzhansky 1937; Mayr 1942; Michel et al. 2010), as parts of a few coevolving “genomic islands” (Turner et al. 2005; Nachman and Payseur 2012; Nosil and Feder 2012), or as free agents establishing locus-specific patterns of variation according to their own particular effects on organismal performance (Dawkins 1976; Wu 2001; Morjan and Rieseberg 2004). In nature, hybrid zones are recognizable because many features of the interacting organisms show concordant, narrow clines, consistent with limited exchange between coadapted gene pools (Key 1968; Harrison 1993; Butlin 1998). Early hybrid zone theory suggested different genes would show different patterns of gene flow and introgression (Barton 1979; Barton and Bengtsson 1986). Barton (1983) and Baird (1995) showed how groups of loci can become synergistically “coupled” (depending on the ratio of selection to recombination) to form strong barriers to gene exchange. With increasingly sophisticated molecular tools for evaluating genome-wide patterns of variation, evidence of genomic heterogeneity in patterns of gene flow is accruing at all levels from locally adapted populations to highly differentiated taxa (e.g., Arnold 2006; Yatabe et al. 2007; Nosil et al. 2008; Fitzpatrick et al. 2009; Gompert et al. 2010).

One promising way to evaluate variation among loci in rates and patterns of gene exchange is statistical analysis of genomic clines. Genomic cline analysis explicitly compares allele or genotype frequencies of each locus (or locus-specific ancestry) to a genome-wide average representing the genomic ancestry of an individual or population (Gompert and Buerkle 2011). The approach was pioneered by Szymura and Barton (1986) as a complementary alternative to geographic cline analysis, where genetic data are evaluated against spatial coordinates or distance. Geographic cline analysis is not always appropriate, for example, in mosaic hybrid zones where hybridizing taxa segregate by habitat at a finer grain than their overlapping geographic ranges (Harrison and Rand 1989; Howard et al. 1993), broadly admixed populations such as humans in North America (Parra et al. 1998), captive livestock herds (Musani et al. 2006), introduced species (Hansen et al. 2001; Fitzpatrick and Shaffer 2007b), or other dynamic hybrid zones with patchy introgression (Macholán et al. 2011).

The basic idea of genomic clines is that, in a hybrid zone or hybrid population between parental populations P1 and P2, each individual (or deme) can be described by their genome-wide mean ancestry *S* = proportion of nucleotides inherited from P1. If all genes followed a single underlying pattern, then *S* is their mutual expected value of locus-specific ancestry: *p*_*i*_ = proportion of copies of locus *i* inherited from P1. Note that shared ancestry is not necessarily equivalent to shared state. For a diagnostic marker (fixed for different alleles in P1 and P2), *p*_*i*_ is the frequency of P1 alleles. For nondiagnostic markers, inferences about ancestry must be made from observations about shared allelic states. The goal of genomic cline analysis is to evaluate locus-specific deviation from the expectations *E*(*p*_i_) = *S*. Particular patterns of deviation can help identify genomic regions experiencing directional selection, hybrid dysfunction, or hybrid vigor (Szymura and Barton 1986; Gompert and Buerkle 2011).

Until now, locus-specific deviations from genome-wide ancestry have been quantified using the polynomial function suggested by Szymura and Barton (1986), and multinomial logistic regression (Lexer et al. 2007; Gompert and Buerkle 2009). Barton's approach is implemented in the package “Analyse” under the name “Concordance” (Barton and Baird 1995). The Barton function has two properties that might be undesirable for genomic clines (Gompert and Buerkle 2011). First, it can be greater than one or less than zero – impossible values for a proportion or probability. Second, even within the interval [0,1], the Barton cline is not necessarily monotonically increasing: The curve can have an intermediate local maximum and minimum, which is unexpected albeit not impossible in light of population genetics theory. Gompert and Buerkle (2011) proposed to overcome these undesirable features by splicing in flat lines in an ad hoc manner. Logistic regression-based approaches satisfy the challenge of modeling probabilities on the interval [0,1], but are less flexible in terms of the form of the fitted curves and assume the independent variable ranges from negative to positive infinity (McCullagh and Nelder 1989). The latter assumption is explicitly violated in genomic cline analysis, where the independent variable is also a proportion on [0,1]. Here, I evaluate two alternative functions that overcome these problems, one phenomenological and the other derived from population genetic theory. I also implement multivariate outlier detection as an alternative to previous hypothesis testing approaches that confound selection and drift (Gompert and Buerkle 2009; Macholán et al. 2011), and simulate hybrid zone and admixture dynamics to assess the effects of different modes of selection.

## Methods

### Deriving the logit-logistic cline model

Bazykin (1969) used the continuous-space diffusion model for population genetics (Fisher 1937) to show that the expected form for a cline caused by heterozygote disadvantage is the familiar logistic function of Richards (1959):


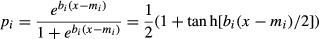
(1a)

or


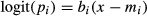
(1b)

where *m*_*i*_ is the cline center for locus *i* (spatial position of the inflection point) and *b*_*i*_ is the slope of the curve at the inflection point, determined in the model by the strength of selection against heterozygotes and the average dispersal distance (Bazykin 1969). Investigators often consider the cline width 1/*b* as a fundamental description of a cline and the same form has been used to describe clines maintained by different kinds of selection or even neutral clines where the cline width depends only on dispersal and the time since secondary contact (Slatkin 1973; Endler 1977; Barton and Gale 1993; Guedj and Guillot 2011). If we assume the genome average ancestry follows a geographic cline


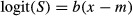
(2)

an expression for *p*_*i*_ in terms of *S* arises on rearranging equation 2 as *x* = (1/*b*) logit(*S*) + *m* and substituting for *x* in equation 1 to get


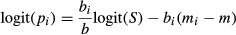
(3)

or, define *u*_*i*_ = *b*_*i*_(*m*_*i*_ − *m*) indicating a relative difference in cline position and *ν*_*i*_ = *b*_*i*_/*b* indicating the relative slope of *p*_*i*_ and


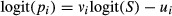
(4)

or


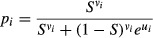
(4b)

This is the “logit-logistic” function describing a genomic cline for a given locus *i* in terms of the average genome-wide ancestry *S*. The parameters can be estimated from data (see below) for a sample of loci as a way to describe multilocus clines and potentially identify exceptional loci, even in situations where the geographic model does not literally apply (e.g., in mosaic hybrid zones or within single hybrid populations) or takes a more complicated form than the simple logistic function, for example, when clines are asymmetric or stepped (Barton 1983; Baird 1995; Porter et al. 1997).

The logit-logistic function follows the constraints appropriate for a relationship between two proportions (*P*_*i*_ ∈ [0,1] and *S* ∈ [0,1]). It also conforms to the definition of *S* as the mean *p*_*i*_, in that when *S* = 0, all *p*_*i*_ = 0, and when *S* = 1, all *p*_*i*_ = 1. The genomic cline for *p*_*i*_ deviates from the mean when the ratio of slopes *ν*_*i*_ deviates from 1.0 and/or the relative cline center *u*_*i*_ differs from zero.

### Alternative cline forms

The Barton cline (Szymura and Barton 1986) relates expected ancestry at a particular locus *p*_*i*_ to genome-wide ancestry *S* as


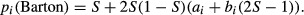
(5)

The coefficients *a*_*i*_ and *b*_*i*_ describe deviations in cline center and steepness relative to perfect concordance (*p*_*i*_ = *S* when *a*_*i*_ = *b*_*i*_ = 0). Locus-specific ancestry *p*_*i*_(Barton) = 0 when genome-wide ancestry *S* = 0 and *p*_*i*_(Barton) = 1 when *S* = 1. The Barton cline is not strictly monotonic (two local extrema are possible when the absolute value of either coefficient is large) and can take values outside [0,1], undesirable for a function describing probabilities. Gompert and Buerkle (2011) introduced ad hoc splicing of flat lines into the function to remove these undesirable features. Given that local extrema might be real features of a given dataset, I adopt the splicing of horizontal lines at 0 and 1, but allow nonmonotonicity in my analysis (as in Barton and Baird 1995). Among the functional forms compared here, the possibility of nonmonotonicity is a unique feature of the Barton function that might make it the best model for certain datasets.

The regularized incomplete beta function is a strictly monotonic, but otherwise very flexible function that also describes the cumulative distribution function of a beta random variable.


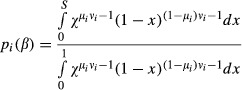
(6)

where *μ* and *ν* are location and shape parameters related to the more familiar beta shape parameters as *α* = *μν* and *β* = (1 − *μ*)*ν* (Kruschke 2010). The *B*(*μ*, *ν*) parameterization is useful for genomic clines because *μ* plays a similar role to *u* in the logit-logistic and *a* in the Barton cline and *ν* a similar role to *v* and *b*. Like the logit-logistic cline, the beta cline is zero when *S* = 0 and one when *S* = 1, it is strictly monotone, and *p*_*i*_(*β*) is never more than one or less than zero.

Although the logit-logistic, Barton, and beta clines have the same number of parameters to estimate, the procrustean splicing of flat lines onto the Barton function to satisfy range constraints makes it more cumbersome than the others in a way not accounted for by model selection criteria such as AIC. That is, more information is required to specify the spliced Barton model even though it does not produce estimates of more parameters (Rissanen 1978, 2005; Grünwald 2004). Moreover, I take it as an underlying principle that the true relationship between locus-specific ancestry probability and the genome-wide average is smooth (continuously differentiable) on the interval (0,1). That is, we do not realistically expect the locus-specific probability to be identically 1.0 except when the genome-wide average is 1.0; and we do not realistically expect an abrupt plateau of identical probabilities over any interval, as implied by the spliced-in flat line. Empirically, the spliced Barton function might be an adequate (or even superior) approximation to the underlying smooth relationship, but all other things being equal, a mathematical model reflecting the underlying principle of smoothness would be preferable.

### Regression-based approaches

Multinomial regression has been used to describe the probability of a locus being heterozygous or homozygous as a function of genome-wide ancestry *S* (Lexer et al. 2007; Gompert and Buerkle 2009). These clines in genotypic frequency differ from clines in allele frequency because multinomial regression captures the heterozygosity component of hybrid genotypes. However, multinomial regression uses sigmoid functions that are somewhat less flexible than the cline models described above, and the estimated probabilities approach zero and one asymptotically instead of reaching limits as *S* reaches its finite limits at zero and one. Despite these limitations, multinomial regression should be considered because of the potential information gained by considering genotype rather than allele frequencies.

A simple binomial (logistic) regression could be used to describe allele frequencies *p*_*i*_ in terms of *S*. This considers the same variables as the cline models above and estimates the same number of parameters. However, it shares with multinomial regression the problem that it is designed to model probabilities that approach zero and one as the independent variable approaches infinity (positive or negative), and therefore is not entirely appropriate for an independent variable on the finite interval [0,1]. From a practical standpoint, this might be desirable flexibility or an undesirable violation of constraints, depending on one's assumptions. That is, if *p*_*i*_ is taken to represent an allele frequency rather than a true ancestry probability, then whatever the value of *p*_*i*_ at *S* = 1 is the predicted frequency of the allele in a “pure” P1 population. However, if ancestry is defined as the expectation (*S* = *E*(*p*_*i*_)) for all *L* loci in the genome, the constraint should not be violated. Approaches for defining and estimating *S* are evolving (e.g., Alexander and Lange 2011; Gompert and Buerkle 2011), and whether the individuals in the admixture analysis are used to define and/or estimate *S* and *p*_*i*_ might affect the validity of one's assumptions.

In the following analysis, I compare the three cline models (logit-logistic, Barton, and beta) and both multinomial and binomial regression in terms of their goodness-of-fit to real and simulated data and their usefulness in identifying outlier loci potentially linked to loci affecting hybrid fitness.

### Fitting models to data

Genetic data are categorical, and often best analyzed as counts. I take the simple approach of considering one allele per locus: *A*_1_ assumed fixed in P1 and absent in P2, so the expected frequency of *A*_1_ in hybrids is the locus-specific ancestry probability *P*. A more general implementation for multiple or nondiagnostic alleles is feasible, but beyond the scope of this paper, which is focused primarily on the value of alternative functional forms.

The genotype of a diploid individual for the particular locus is represented as a count (number of *A*_1_ alleles: 0, 1, or 2) and can be modeled as a random draw of two from a binomial distribution with probability of success *P*. Likewise, a sample of *n* individuals can be modeled as a random draw of 2*n* from the same binomial distribution. Let *x* be the number of *A*_1_ alleles in a sample of size 2*n*. The probability of *x* given *P* is the binomial mass 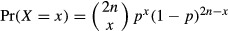. Under Hardy–Weinberg assumptions (e.g., Hardy 1908; Hartl and Clark 1997), *P* is the population allele frequency of *A*_1_. But population-level Hardy–Weinberg equilibrium need not be assumed if *P* can be estimated independently for an individual or group. The genomic clines approach models variation in *P* among individuals, groups, or sites (e.g., eqs. 1–3), such that the binomial assumption need apply only “locally” for individuals characterized by the same *S*.

Given *K* samples with varying *P*, the likelihood of the parameters is proportional to the product of binomials:


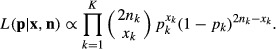
(7)

Now let *p*_*k*_ be given by one of the cline functions (eqs. 4, 5, or 6). Then equation 7 can be used to calculate the likelihood of model coefficients given *x*, *n*, and an estimate of *S* for each sample. Note that this is the same likelihood function maximized in binomial regression, with logit = (*p*_*k*_) = *α* + *βS* (McCullagh and Nelder 1989).

I wrote functions in R (R Development Core Team 2011) to search for maximum likelihood estimates for each function described here. I implemented the likelihood search using the native R function “optim.” For the regression-based methods, I used the functions “multinom” and “glm” to fit multinomial and binomial models, respectively (Venables and Ripley 2002).

Gompert and Buerkle (2011) developed a hierarchical Bayesian method for estimating Barton cline parameters while accounting for uncertainty in parental allele frequencies and explicitly modeling locus-specific effects. This Bayesian method has been extended to account for linkage relationships and uncertainty in genotyping (Gompert and Buerkle 2012; Gompert et al. 2012a,b). Presumably, their approach could readily incorporate the cline functions reviewed here, but developing such computational tools is beyond the scope of this study. My goal is to illustrate the value of considering multiple alternatives for genomic cline analysis. I presume that the relative performance of the functional forms under the likelihood framework predicts relative performance under a Bayesian framework because the additional uncertainty accounted for (owing to genotyping error and uncertainty in parental allele frequency estimates) would be identical, no matter which equation is being fit. This might not be true in the case of the multinomial, which relies on accurate discrimination of heterozygotes and homozygotes; genotype frequency estimates might be prone to greater error than allele frequency estimates.

### Outlier detection

The major goal of genomic cline analysis is usually to identify markers affected by selection – that is, those linked to genes contributing to hybrid dysfunction, hybrid vigor, or local adaptation (e.g., Lexer et al. 2007; Gompert et al. 2012a). However, it is insufficient to identify candidate markers by rejecting the naïve null hypothesis that locus-specific ancestry should match the genome-wide average (*p*_*i*_ = *S*). This is because genetic drift alone will generate real variation among loci. This problem has been acknowledged by previous investigators (e.g., Gompert and Buerkle 2009). But a generally acceptable solution remains elusive.

Traditional hypothesis tests based on likelihood (Barton and Baird 1995; Macholán et al. 2011) or randomization (Gompert and Buerkle 2009) consider whether a model fitted to a given locus deviates from the null hypothesis more than expected from sampling alone. But to identify markers that differ from the average by more than expected from *drift and sampling*, we need to assess how the true distribution of parameters might be affected by drift. It appears that no appropriate theory has been described for genomic clines, but it would likely depend on difficult to measure factors such as population size, dispersal behavior, and time since secondary contact (Hartl and Clark 1997). Barton (2008) and Polechova and Barton (2011) provide some relevant theory for geographic clines, but no theory-based test for a null distribution of cline parameters has been proposed.

Long (1991) provides a sample-wide test for heterogeneity among markers for the special case of a single admixed population (see Fitzpatrick et al. 2009). Gompert and Buerkle (2011) identified outliers by assuming univariate normal distributions for each parameter of the Barton cline. This approach was far less prone to false positives than the methods in INTROGRESS, which test the naïve null hypothesis *p*_*i*_ = *S*, with no effect of drift (Gompert and Buerkle 2010, 2012). I take a similar (but multivariate) statistical approach here, using a traditional, general-purpose method to identify outliers in multivariate data.

Assuming the joint distribution of parameter estimates among loci is multivariate normal, the squared Mahalanobis distance *D*^2^ of each locus is expected to be distributed as a *χ*^2^ random variable with degrees of freedom equal to the number of parameters (Johnson and Wichern 1998). A locus with *D*^2^ greater than a specified critical value or visually deviating from a quantile–quantile plot can be declared a statistical outlier (Johnson and Wichern 1998). For automated outlier detection, I used the Bonferroni-adjusted critical *P*-value, but visual inspection of quantile–quantile plots might be the best recommendation for exploratory analysis of real data.

This approach (or other general-purpose multivariate methods) relies on the observed variation among markers to establish an empirical basis for outlier detection. The advantage is that the distribution evolves over time with genetic drift. So, while genetic drift should make it progressively easier to reject the naïve null hypothesis for any neutral marker with each passing generation, multivariate outlier detection should remain more robust.

### Simulated data

To examine how well the cline models and regression-based methods fit data and reveal outliers, I used stochastic individual-based simulations of secondary contact between two populations under two kinds of population structure. First is a geographically structured hybrid zone where we can use decades of research on geographic clines to inform our expectations with respect to genomic clines. Second is a single admixed population with random mating where geographic clines are not relevant. For each of these scenarios, I consider the effects of immigration from pure parental populations and several kinds of locus-specific selection.

For all model runs, secondary contact was initiated as 500 individuals from parental population P1 on one half of the modeled space, and 500 individuals from P2 on the other half. I kept track of 100 unlinked loci with two alleles (a diagnostic, fixed difference between P1 and P2). Most loci were neutral, but up to four could influence hybrid fitness. These conditions represent “low coupling” in the sense that synergistic effects of many loci are not possible. If many loci affect fitness, we should expect stepped clines and less clear distinction between “normal” and “outlier” markers.

#### Hybrid zone model

Full details and computer code (written in R) are provided in the publicly available R-package “HIest”. Here, I describe the model verbally and explain the range of parameter values investigated. The hybrid zone is modeled as a rectangle in which a diploid individual can occupy any *x–y* coordinate (space is continuous, 2-dimensional). Individuals are outcrossing hermaphrodites and act as the female parent of a random number of offspring drawn from a Poisson distribution with expected value determined by local density dependence and genetics. Individuals that draw a number greater than 0 draw a mate at random from all other individuals in the population according to a normal density function of their distance from the focal individual. Each offspring draws its *x–y* location from a bivariate normal distribution centered on its mother. For all simulations analyzed here, space ranged from −3 to +3 in both dimensions and both the dispersal and mating curves had standard deviations of 0.3 (5% of the space).

In the absence of selection, the number of offspring mothered by each individual is a Poisson random variable with expected value given by a Beverton–Holt function (Begon et al. 2006) of local density measured as the sum over all individuals weighted by a normal density function of their distance from the focal individual with standard deviation 0.2. For most simulations I used a baseline growth rate *R* = 2 and local carrying capacity *K* = 14, which resulted in a steady state of approximately 1000 individuals distributed evenly across space.

In the presence of selection, the Beverton–Holt function was simply multiplied by the individual's relative fitness (see below). This means selection was limited to the female component of fitness, effectively weakening the intensity of selection.

The hybrid zone could be closed or open. When closed, if an offspring drew a location outside of the defined space, it was reassigned to a position at the nearest edge. That is boundaries were reflecting, and no immigrants were added during the simulation. When open, if an offspring drew a location within 5% of either *x*-boundary, it was replaced by a pure parental genotype (P1 on one side, P2 on the other). The *y*-boundary was still reflecting. Thus, for an open simulation, an average of about 5% of the individuals in the hybrid zone on each side (those closest to the ends) were replaced by immigrants from the parental populations.

#### Admixture model

To maintain as much similarity between models as possible, I simulated a panmictic population using the same continuous space and local density-dependent reproduction, but mates were chosen at random from all other individuals in the population without regard to distance, and offspring drew their *x–y* coordinates from a uniform distribution covering the entire space. That is, there was no spatial correlation between mates or between parents and offspring. As with the hybrid zone model, the population could be closed or open. Again, in the latter case, an average of about 5% of the individuals were replaced by immigrants from each parental population.

#### Hybrid fitness

I modeled four kinds of locus-specific fitness effects. One locus could affect fitness according to an environmental gradient, one could have heterozygote disadvantage or advantage, and a pair of loci could have a Dobzhansky–Muller incompatibility (Turelli and Orr 2000; Fitzpatrick 2008). The remaining 96 loci were always neutral.

The environmental phenotype *z* of an individual was determined by one locus (the “E locus”) and could take values of 1.0, 0.5, or 0.0 for P1 homozygotes, heterozygotes, and P2 homozygotes, respectively. The environmental fitness component for an individual was calculated as a Gaussian function of the difference between its phenotype and the value *g* of the environmental gradient


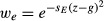
(8)

where *s*_*E*_ determines the strength of selection (I used values of 2 or 4). This fitness function gives P2 homozygotes the advantage where *g* = 0, heterozygotes the advantage where *g* = 0.5, and P1 homozygotes the advantage where *g* = 1. The environmental gradient was defined as a logistic function of the x-dimension (logit(*g*) = *b*(*x*−*m*)) with a slope *b* of 1 and midpoint *m* at 1, 2, or -4 from the hybrid zone center (the latter case amounting to a universal advantage for the P1 allele). In the admixture model, the environmental gradient (with midpoints at 1 or 2) favored P2 alleles because most of the area was favorable to P2 homozygous phenotypes.

Heterozygote advantage was modeled by assigning fitnesses to genotypes of an “H” locus:


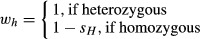
(9)

I used *s*_*H*_ = 0.4 and *s*_*H*_ = 0.8. Heterozygote disadvantage was modeled by


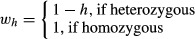
(10)

with *h* = 0.4 or *h* = 0.8.

Finally, two other loci (*A* and *B*) could have a Dobzhansky–Muller incompatibility with fitness according to Table 1. I used only the simple case of a partial recessive incompatibility with selection *d* = 0.4 or 0.8. Simulations were performed with no selection, one kind of selection, or combinations of environmental selection, heterozygote dysfunction, and Dobzhansky–Muller incompatibilities (Table 2).

**Table 1:** Fitness effects of a Dobzhansky–Muller incompatibility between loci *A* and *B*. Subscripts denote alleles from P1 or P2

	*A*_1_*A*_1_	*A*_1_*A*_2_	*A*_2_*A*_2_
*B*_1_*B*_1_	1	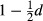	1−*d*
*B*_1_*B*_2_	1	1	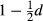
*B*_1_*B*_2_	1	1	1

This is a simple case of the general model (Turelli and Orr 2000; Gavrilets 2004; Fitzpatrick 2008) with the incompatibility acting as a partial recessive.

**Table 2:** Parameter values for simulations

*b*	*s*_*E*_	*m*	*H*	*s*_*H*_	*d*
0	0	0	0	0	0
1	2	1	0	0	0
1	2	2	0	0	0
1	4	1	0	0	0
1	4	2	0	0	0
0	0	0	0.4	0	0
0	0	0	0.8	0	0
0	0	0	0	0	0.4
0	0	0	0	0	0.8
1	2	1	0.4	0	0
1	2	2	0.4	0	0
1	2	1	0	0	0.4
1	2	2	0	0	0.4
0	0	0	0.4	0	0.4
1	2	1	0.4	0	0.4
1	2	2	0.4	0	0.4
0	0	0	0	0.8	0
2	4	−4	0	0	0

Each set was run under closed and open conditions for both the hybrid zone and admixture models (for a total of 72 runs). *b* and *m* are the slope and midpoint of the environmental gradient; *s*_*E*_ is the strength of selection on the environmental phenotype (eq. 8); *h* is heterozygote disadvantage (eq. 10); *s*_*H*_ is heterozygote advantage (eq. 9); *d* is the strength of the Dobzhansky–Muller incompatibility (Table 1).

**Figure 1:**
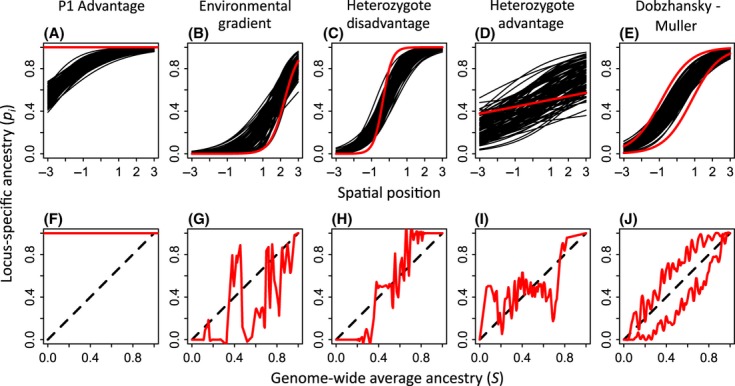
Example simulation from the open hybrid zone model with different kinds of single-locus selection. The top row of panels shows spatial clines (eq. 1) for 99 neutral loci (black lines) and one selected locus (red). The second row shows cubic smoothing splines fitted to the selected locus data as a function of genome-wide ancestry. In the P1 advantage case (A and F), relative female fitness of P2 homozygotes was 0.2 and heterozygotes 0.6. The environmental gradient (B and G) was centered at spatial position 2 and the strength of selection was *s* = 4 (eq. 8). Heterozygote disadvantage (C and H) was *h* = 0.8 and disadvantage (D and I) *s* = 0.8. The Dobzhansky–Muller incompatibility was determined by *d* = 0.8. In each case, the simulation was run for 50 generations (results are similar at 25 and 100 generations).

**Figure 2:**
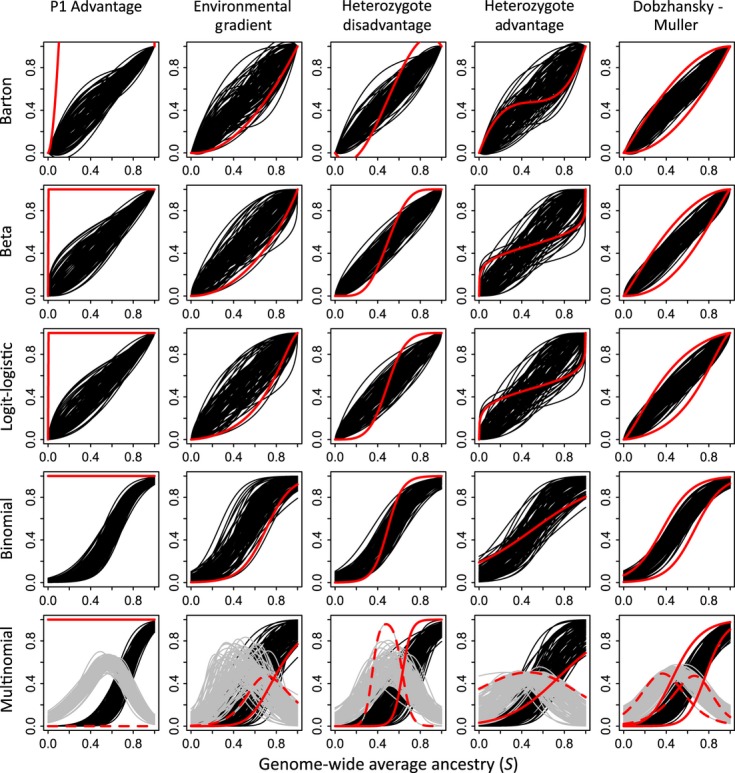
Genomic clines fitted to simulated data after 50 generations in the open hybrid zone model (same simulations as Fig. 1). Black lines illustrate unlinked neutral markers; red lines illustrate selected markers.

**Figure 3:**
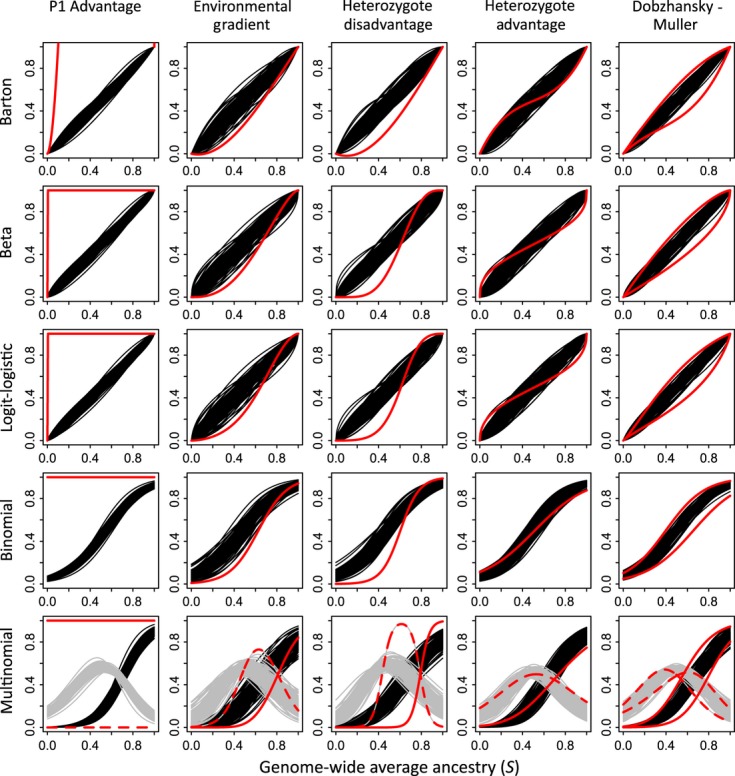
Genomic clines fitted to simulated data after 50 generations in the open admixture model (otherwise same conditions as Figs. 1 and 2). Black lines illustrate unlinked neutral markers; red lines illustrate selected markers.

These different causes of hybrid fitness variation can lead to distinct patterns in geographic and genomic clines (Fig. 1). Environmental selection (or universal advantage of an allele from one parental lineage) can cause geographic and genomic clines to be displaced or result in fixation of one allele (Fig. 1A, B, F, G). Heterozygote disadvantage can cause a locus-specific cline to be steeper than average in both geographic and genomic analyses (Fig. 1C, H). Heterozygote advantage causes a relatively shallow geographic cline and an “inside-out” genomic cline (Fig. 1D, I). Dobzhansky–Muller incompatibilities tend to result in displacement of each partner locus in opposite directions in both kinds of analysis (Fig. 1E, J). The same kinds of genomic cline patterns arise under the same kinds of fitness models whether geographic structure is present or not (hybrid zone vs. admixture model), but the expected variance among neutral markers was higher in the hybrid zone model (Figs. 2 and 3).

The examples depicted in Figure 1 also illustrate the hitchhiking effect of each kind of selection; at least for the strong selection simulated here, spatial clines for the unlinked neutral loci were strongly distorted relative to the no-selection case, except for the Dobzhansky–Muller incompatibility case, for which the neutral clines (black lines in Fig. 1E) are indistinguishable from the no-selection case (not shown).

### An empirical example

**Figure 4:**
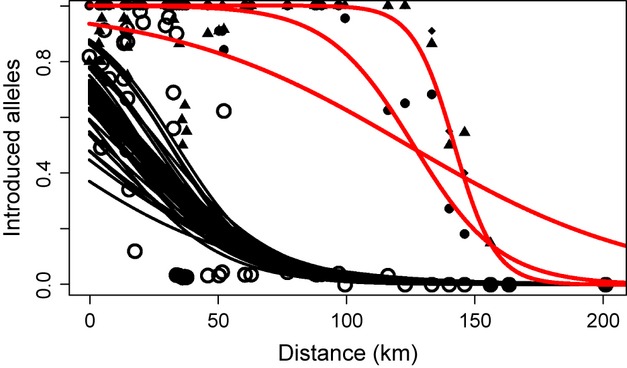
Geographic cline analysis: Sigmoid clines fitted to introduced allele frequencies at 67 diagnostic markers in the California tiger salamander hybrid zone (Fitzpatrick et al. 2010). Red lines and black symbols represent “superinvasive” markers. Open circles show the genome wide average ancestry at each sample site. Most markers do not asymptote to 1.0 on the left side because there is no “pure” Barred Tiger Salamander region in California; they were introduced patchily into native populations of the California Tiger Salamander in the Salinas Valley.

To illustrate analysis of a real dataset, I used published data from an expanding hybrid swarm formed in the 1940's when Barred Tiger Salamanders (*Ambystoma tigrinum mavortium*) from Texas were introduced into California and encountered the native California Tiger Salamander, *A. californiense* (Fitzpatrick and Shaffer 2007b; Fitzpatrick et al. 2010). The dataset includes 773 salamanders from 58 sites scored for 67 nuclear SNPs. Two of these SNPs are “ringers” having no heterozygotes in the dataset because of technical problems with genotype scoring; they are included here to assess the visibility of heterozygote deficits in genomic cline analyses. Fitzpatrick et al. (2010) showed strong evidence of genomic heterogeneity, with three markers having introgressed 95 km further into the native range than the rest (Fig. 4). Although this striking pattern is hard to miss, overall the dataset is not well suited to geographic cline analysis because there is an abrupt transition from a hybrid swarm in the Salinas Valley, where breeding sites vary widely in mean *S* without much relationship to geographic distance, to essentially pure native populations outside the Salinas Valley. That is the biological situation is not well represented as a cline or series of clines between geographic ranges of two parental forms. For similar reasons, Macholán et al. (2011) used Barton's concordance, INTROGRESS, and GENELAND to study patchy, long distance introgression in the house mouse hybrid zone. Genomic cline analysis offers a satisfying alternative with potential to reveal important patterns of variation that have not sorted out along a geographic gradient.

The data consist of individual diploid genotypes of markers presumed to be diagnostic (based on analyses of reference populations). Hybrid *Ambystoma* can have 0, 1, or 2 introduced (*A. t. mavortium*) alleles. Analysis of nondiagnostic markers will require an additional step relating observed genotypes to allelic ancestry*p*_*i*_ (Gompert and Buerkle 2011).

I analyzed the data using the R-package INTROGRESS (Gompert and Buerkle 2010), using their randomization test (assuming negligible genetic drift since secondary contact) to identify markers potentially linked to loci affecting hybrid fitness. I also used the stand-alone program bgc (Gompert and Buerkle 2012) to fit the spliced Barton function and identify outliers using Gompert and Buerkle's (2011) univariate Bayesian method. Then I fit each of the cline and regression models described here and used multivariate outlier detection to identify candidate markers. Fitting was done using allele or genotype counts and genome-wide average *S* per locality to account for nonindependence of salamanders sampled from the same breeding pond. Given the likely importance of genetic drift over the past 60 years (20–30 generations), I predicted that the outlier detection methods would be more conservative than INTROGRESS.

## Results

### Simulated data

#### Testing the naïve null hypothesis

As expected for data influenced by genetic drift, the naïve null hypothesis (*p*_*i*_ = *S*) was relatively easy to reject for neutral markers (Table 3). The parametric test in INTROGRESS (Gompert and Buerkle 2009, 2010) flagged 99 to 100% of the markers in no-selection simulations as significantly deviating from the null hypothesis (Bonferroni-adjusted critical *P* ≤ 0.05/100 = 0.0005), while their permutation test was somewhat more conservative. For comparison, I also tested the naïve null hypothesis using traditional likelihood ratio tests for the fitted cline models; results were virtually identical to the Gompert and Buerkle (2009) permutation test (Table 3). As expected, detection rates increased with the influence of drift over time and in closed populations (Table 3). These results strongly caution against naïve null hypothesis tests (assuming zero drift) for identifying candidate markers. As pointed out by Long (1991) in a similar context, the effects of selection and drift are confounded in these tests.

**Table 3:** Numbers of neutral loci (out of 100) deviating from the naive null hypothesis (no-selection simulations only)

Simulation info.				INTROGRESS		Cline models		
		
Str.	Imm.	Time	N	Param.	Permut.	Barton	Beta	Logit-logistic
AD	Closed	10	935	99	23	26	26	26
AD	Closed	25	1010	99	52	53	53	53
AD	Closed	50	954	99	65	65	65	65
AD	Closed	100	1018	100	74	73	73	73
AD	Open	10	959	100	14	13	13	13
AD	Open	25	957	100	22	21	22	21
AD	Open	50	990	100	22	18	19	19
AD	Open	100	966	100	27	23	23	23
HZ	Closed	10	975	100	25	25	26	28
HZ	Closed	25	986	100	56	58	62	60
HZ	Closed	50	966	100	81	84	84	84
HZ	Closed	100	1049	100	89	86	86	86
HZ	Open	10	963	100	26	26	24	26
HZ	Open	25	1014	100	52	49	51	48
HZ	Open	50	1060	100	58	63	60	60
HZ	Open	100	966	100	72	69	67	68

“Str.” is either the hybrid zone (HZ) or admixture (AD) model, “Imm.” is either closed or open to immigration from pure parental populations, Time is the number of elapsed generations since secondary contact, *N* is the population size at the time of census, “Param.” and “Permut.” refer to the parametric and permutation-based hypothesis tests in INTROGRESS (Gompert and Buerkle 2009, 2010). Numbers under the cline models are counts of significant likelihood ratio tests of fitted models versus *H*_0_:*p*_*i*_ = *S*. Critical values for all tests were Bonferroni adjusted (*P* ≤ 0.05/100 = 0.0005).

#### Goodness-of-fit

Comparing models strictly in terms of how well the fitted curves correspond to the data, the Barton and beta clines were often best (Table 4), but multinomial or binomial regression were sometimes better in closed population simulations. This is probably explained by the lack of “pure” (*S* = 0 or 1) individuals anchoring the curves at each end of the ancestry spectrum. Cline models always fit best in open populations. The relative goodness-of-fit tended to change over time since secondary contact, especially in the hybrid zone scenario, where the beta cline fit best early on, but the Barton cline fit best in later generations when the hybrid zone was open to immigration, and multinomial regression fit best in later generations when the hybrid zone was closed (Table 4). Representative examples are illustrated in Figures 2 and 3.

**Table 4:** Goodness-of-fit of alternative models to simulated genomic clines

Time	Immigration	Structure	Multinomial	Binomial	Logit-logistic	Beta	Barton
10	Closed	AD	19.22	34.17	7.83	12.67	26.11
25	Closed	AD	18.56	33.67	8.78	13.89	24.89
50	Closed	AD	18.44	30.17	8.94	14.89	26.28
100	Closed	AD	18.89	26.94	10.28	14.61	25.72
10	Open	AD	10.56	0.28	21.67	30.06	37.44
25	Open	AD	10.56	0.39	20.67	30.61	37.78
50	Open	AD	9.44	0.11	22.61	29.33	38.50
100	Open	AD	10.22	0.67	22.33	30.89	35.89
10	Closed	HZ	6.28	1.06	18.28	43.78	30.61
25	Closed	HZ	12.06	4.83	16.11	39.17	27.78
50	Closed	HZ	20.06	12.72	12.39	28.17	26.06
100	Closed	HZ	31.78	11.94	9.83	16.67	26.44
10	Open	HZ	6.67	0.06	21.50	40.06	31.72
25	Open	HZ	3.50	0.67	21.28	40.94	33.61
50	Open	HZ	2.33	0.50	23.78	36.72	36.67
100	Open	HZ	2.39	0.44	23.56	35.22	38.39

Each number is the average percent of loci best fit by each model (according to *AIC*_*c*_) across all simulations. Time is the number of elapsed generations since secondary contact, “Immigration” is either closed or open to immigration from pure parental populations, and “Structure” is either the hybrid zone (HZ) or admixture (AD) model.

#### Outlier detection

On the other hand, comparing models in terms of how well they expose exceptional loci, the logit-logistic had the best combination of precision (best ratio of true positive to false positive results) and sensitivity (Table 5). The Barton cline had the lowest sensitivity (it missed more selected loci than any other model) and multinomial regression had rather high false-positive rates (low precision).

**Table 5:** Precision and sensitivity of outlier detection based on alternative models fitted to simulated data

Model	Correct	Missed	False pos	Precision	Sensitivity
Barton	52	572	43	0.55	0.08
Beta	94	530	89	0.51	0.15
llogit	118	506	43	0.73	0.19
Logistic	117	507	57	0.67	0.19
Multinom	126	498	199	0.39	0.20

Tabulations include all simulations, each evaluated at four times (10, 25, 50, and 100 generations). The total number of selected loci (Correct + Missed = 624) includes all forms of selection across all simulations (Table 2).

**Figure 5:**
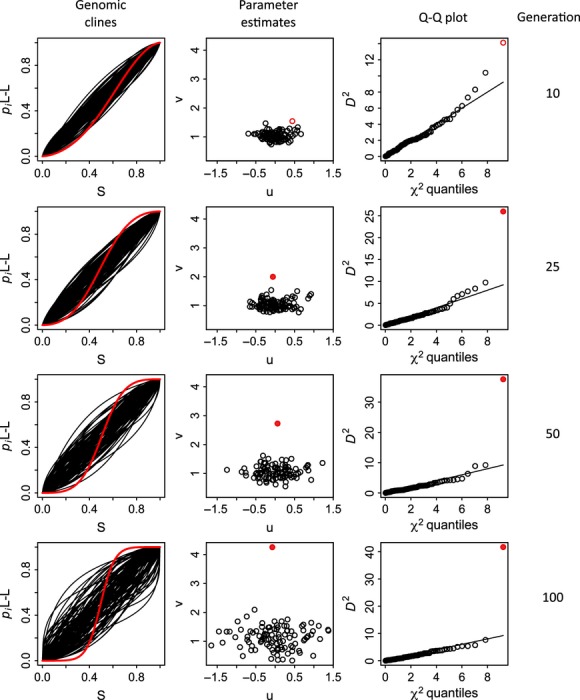
Effects of genetic drift on genomic cline parameters and multivariate outlier detection. Data are from a simulation of the closed hybrid zone model with strong heterozygote disadvantage (*h* = 0.8). Black lines and symbols represent 99 unlinked neutral markers; red represents the selected marker. The fitted logit-logistic model is shown, but results were similar for other cline models. The selected locus was a statistically significant outlier in generations 25, 50, and 100. There were no significant outliers in generation 10 (*α* = 0.0005). The line in each *Q*–*Q* plot illustrates the expectation of equality between empirical quantiles of the mahalanobis distance (*D*^2^) and quantiles of the *χ*^2^ distribution with 2 degrees of freedom.

The impact of genetic drift on cline parameters and the robustness of multivariate outlier detection are illustrated in Figure 5. As expected in a closed hybrid zone, locus-specific clines become increasingly variable over time. Because outlier detection uses the empirical distribution of parameter estimates, variation owing to drift alone did not result in statistical outliers in this example. In the example (Fig. 5), there are many loci at generations 50 and 100 that would be outliers if compared to the distribution of clines at generations 10 or 25, but appear statistically normal in their proper contexts.

### Ambystoma hybrid swarm

**Figure 6:**
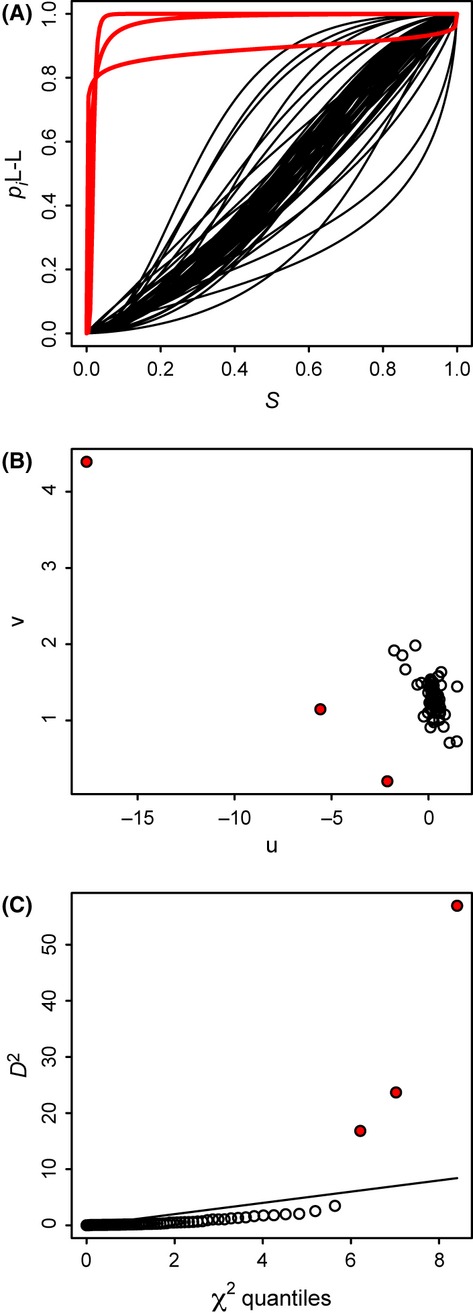
Logit-logistic clines fitted to the tiger salamander data (A), and multivariate outlier detection (B and C). Red lines and symbols represent the three “superinvasive” markers (Fitzpatrick et al. 2010), which are the only statistical outliers.

The permutation test from INTROGRESS flagged 65 of 67 markers as deviating from the naïve null hypothesis (Bonferroni adjusted critical *P* ≤ 0.05/67 = 0.00075). Multivariate outlier detection using the fitted binomial regression, logit-logistic cline, and beta cline models flagged only the previously identified “superinvasive” markers (cm6E11, cm12C11, cm23C6 Fig. 6). Analyses with the Barton cline (using the Bayesian method of bgc or my likelihood implementation in Hiest) failed to detect cm12C11 as an outlier. Outlier detection using multinomial regression identified the three superinvasive markers and the two “ringers” with no heterozygous genotypes. Although the latter detections add nothing to our biological knowledge of the example system, they do underscore the unique ability of the multinomial regression approach to describe genotype frequency variation.

## Discussion

Comparative analysis of genomic clines yields important insights into hybrid zones, admixture dynamics, and genomic heterogeneity, particularly when geographic clines are not applicable. Although the genomic cline approach was first proposed over 25 years ago (Szymura and Barton 1986), the approach has suffered from a lack of alternative models, little information on expected signatures of different kinds of selection, and no rigorous method for identifying exceptional markers while accounting for genetic drift (until Gompert and Buerkle 2011, 2012). I have addressed these problems by introducing the beta and logit-logistic cline models, simulating hybrid zone and admixture dynamics to investigate the effects of different kinds of selection on genomic cline shape, and implementing a well-known multivariate outlier detection method (similar to Gompert and Buerkle's (2011) univariate approach). At least for the conditions examined here, the logit-logistic cline model is the best for identifying markers of interest.

The beta and logit-logistic cline models, introduced for the first time here, overcome some theoretical shortcomings of previous approaches. In particular, they appropriately model the relationship between two proportions or probabilities (variables defined only on the finite interval [0,1]) and meet the constraint (imposed by the definition of genomic ancestry) that ancestry probability for a given locus must be zero or one (respectively) when the genome-wide ancestry probability is zero or one (respectively). The Barton cline model meets the latter constraint, but allowed the dependent variable to trespass above one or below zero without ad hoc truncation (Gompert and Buerkle 2011). Multinomial regression properly models a dependent variable on [0,1], but assumes the independent variable can stretch from negative to positive infinity.

Although beta distributions can be used to model allele frequency variation in structured populations (Balding and Nichols 1995; Pritchard and Donnelly 2001), the beta cline is phenomenological, as are the Barton cline and regression approaches. In contrast, the logit-logistic cline function arises from simple population genetic theory for geographic clines (Bazykin 1969). It is interesting that the logit-logistic was not commonly the best model for describing data in terms of goodness-of-fit (Table 4). Perhaps this is not surprising given that the simple geographically sigmoid model from which it was derived is inaccurate when several loci affect fitness in the center of a hybrid zone (Barton 1983; Baird 1995). In particular, when several linked loci affect fitness, there is a “coupling” effect where multilocus clines are steeper and more coincident in the center of a hybrid zone (Barton 1983; Baird 1995). This has been described as a “step,” where the logistic function describing cline shape in the cline center is discontinuous with more gradual “tails of introgression” on either side (Barton and Bengtsson 1986; Barton and Gale 1993). Further development is needed to treat this, perhaps more realistic model. However, as a practical matter, the logit-logistic was most effective for identifying outliers caused by natural selection in my simulations (Table 5).

As for geographic clines, there is no one-to-one correspondence between genomic cline shape and mode of selection. For example, previous work showed that whether heterozygote disadvantage or epistatic hybrid dysfunction reliably cause sigmoid genomic clines depends on population structure (the influence of dispersal and drift) in addition to selection intensity (e.g., Gompert and Buerkle 2011; Gompert et al. 2012b). A few qualitative generalizations are supported by those studies and the present results. Selection against heterozygotes tends to steepen genomic clines, while heterozygote advantage flattens them out. Dobzhansky–Muller incompatibilities tend to generate complementarily displaced pairs of clines. These might be impossible to distinguish from displacement caused by directional selection or an offset environmental gradient. As noted for geographic clines (Barton and Gale 1993; Kruuk et al. 1999), an environmental gradient can generate clinal patterns indistinguishable from environment-independent selection against heterozygotes. Finally, genomic cline analysis is inherently relativistic; if many markers are associated with fitness in similar ways, they will not be seen as statistical outliers. Nevertheless, genomic clines provide an excellent way to screen for exceptional loci, and joint consideration of alternative cline forms offers valuable perspective on hybrid zone dynamics and patterns of genomic heterogeneity. Future work should incorporate these alternative functional forms with methods accounting for uncertainty associated with nondiagnostic markers.

## References

[b1] Alexander DH, Lange K (2011). Enhancements to the admixture algorithm for individual ancestry estimation. BMC Bioinformatics.

[b2] Anderson E (1948). Hybridization of the habitat. Evolution.

[b3] Anderson E, Stebbins GL (1954). Hybridization as an evolutionary stimulus. Evolution.

[b4] Arnold M (2006). Evolution Through Genetic Exchange.

[b5] Arnold ML, Martin NH (2009). Adaptation by introgression. J. Biol.

[b6] Baird SJE (1995). A simulation study of multilocus clines. Evolution.

[b7] Balding DJ, Nichols RA (1995). A method for quantifying differentiation between populations at multi-allelic loci and its implications for investigating identity and paternity. Genetica.

[b8] Barton NH (1979). Gene flow past a cline. Heredity.

[b9] Barton NH (1983). Multilocus clines. Evolution.

[b10] Barton NH (2008). The effect of a barrier to gene flow on patterns of geographic variation. Genet. Res.

[b11] Barton NH, Baird SJE (1995). Analyse- an application for analysing hybrid zones.

[b12] Barton NH, Bengtsson BO (1986). The barrier to genetic exchange between hybridizing populations. Heredity.

[b13] Barton NH, Gale KS, Harrison RG (1993). Genetic analysis of hybrid zones. Hybrid zones and the evolutionary process.

[b14] Bazykin AD (1969). Hypothetical mechanism of speciation. Evolution.

[b15] Begon M, Townsend CR, Harper JL (2006). Ecology: from individuals to ecosystems.

[b16] Buerkle CA, Lexer C (2008). Admixture as the basis for genetic mapping. Trends Ecol. Evol.

[b17] Butlin R, Howard DJ, Berlocher SH (1998). What do hybrid zones in general, and the chorthippus parallelus zone in particular, tell us about speciation?. Endless forms: species and speciation.

[b18] Dawkins R (1976). The selfish gene.

[b19] Dobzhansky T (1937). Genetics and the origin of species.

[b20] Endler JA (1977). Geographic variation, speciation, and clines.

[b21] Fisher RA (1937). The wave of advance of advantageous genes. Ann. Eugen.

[b22] Fitzpatrick BM (2008). Hybrid dysfunction: population genetic and quantitative genetic perspectives. Am. Nat.

[b23] Fitzpatrick BM, Shaffer HB (2007a). Hybrid vigor between native and introduced salamanders raises new challenges for conservation. Proc. Natl Acad. Sci. USA.

[b24] Fitzpatrick BM, Shaffer HB (2007b). Introduction history and habitat variation explain the landscape genetics of hybrid tiger salamanders. Ecol. Appl.

[b25] Fitzpatrick BM, Johnson JR, Kump DK, Shaffer HB, Smith JJ, Voss SR (2009). Rapid fixation of non-native alleles revealed by genome-wide snp analysis of hybrid tiger salamanders. BMC Evol. Biol.

[b26] Fitzpatrick BM, Johnson JR, Kump DK, Smith JJ, Voss SR, Shaffer HB (2010). Rapid spread of invasive genes into a threatened native species. Proc. Natl Acad. Sci. USA.

[b27] Gavrilets S (2004). Fitness landscapes and the origin of species.

[b28] Gompert Z, Buerkle CA (2009). A powerful regression-based method for admixture mapping of isolation across the genome of hybrids. Mol. Ecol.

[b29] Gompert Z, Buerkle CA (2010). Introgress: a software package for mapping components of isolation in hybrids. Mol. Ecol. Resour.

[b30] Gompert Z, Buerkle CA (2011). Bayesian estimation of genomic clines. Mol. Ecol.

[b31] Gompert Z, Buerkle CA (2012). bgc: software for bayesian estimation of genomic clines. Mol. Ecol. Resour.

[b32] Gompert Z, Fordyce JA, Forister ML, Shapiro AM, Nice CC (2006). Homoploid hybrid speciation in an extreme habitat. Science.

[b33] Gompert Z, Forister ML, Fordyce JA, Nice CC, Williamson RJ, Buerkle CA (2010). Bayesian analysis of molecular variance in pyrosequences quantifies population genetic structure across the genome of *Lycaeides* butterflies. Mol. Ecol.

[b34] Gompert Z, Lucas LK, Nice CC, Fordyce JA, Forister ML, Buerkle CA (2012a). Genomic regions with a history of divergent selection affect fitness of hybrids between two butterfly species. Evolution.

[b35] Gompert Z, Parchman TL, Buerkle CA (2012b). Genomics of isolation in hybrids. Philos. Trans. R. Soc. Lond. B Biol. Sci.

[b36] Grünwald P, Grünwald P, Myung I, Pitt M (2004). Tutorial on minimum description length. Advances in minimum description length: theory and applications.

[b37] Guedj B, Guillot G (2011). Estimating the location and shape of hybrid zones. Mol. Ecol. Resour.

[b38] Hansen MM, Nielsen EE, Bekkevold D, Mensberg K-LD (2001). Admixture analysis and stocking impact assessment in brown trout (salmo trutta), estimated with incomplete baseline data. Can. J. Fish. Aquat. Sci.

[b39] Hardy GH (1908). Mendelian proportions in a mixed population. Science.

[b40] Harrison RG (1990). Hybrid zones: windows on evolutionary process. Oxf. Surv. Evol. Biol.

[b41] Harrison RG, Harrison RG (1993). Hybrids and hybrid zones: historical perspective. Hybrid zones and the evolutionary process.

[b42] Harrison RG, Rand DM, Otte D, Endler JA (1989). Mosaic hybrid zones and the nature of species boundaries. Speciation and its consequences.

[b43] Hartl DL, Clark AG (1997). Principles of population genetics.

[b44] Hewitt GM (1988). Hybrid zones - natural laboratories for evolutionary studies. Trends Ecol. Evol.

[b45] Hewitt GM (2001). Speciation, hybrid zones and phylogeography - or seeing genes in space and time. Mol. Ecol.

[b46] Howard DJ, Waring GL, Tibbets CA, Gregory PG (1993). Survival of hybrids in a mosaic hybrid zone. Evolution.

[b47] Johnson R, Wichern DW (1998). Applied multivariate statistical analysis.

[b48] Key KHL (1968). the concept of stasipatric speciation. Syst. Zool.

[b49] Kruschke JK (2010). Doing Bayesian data analysis: a tutorial with R and BUGS.

[b50] Kruuk LEB, Baird SJE, Gale KS, Barton NH (1999). A comparison of multilocus clines maintained by environmental adaptation or by selection against hybrids. Genetics.

[b51] Lexer C, Buerkle CA, Joseph JA, Heinze B, Fay MF (2007). Admixture in european populus hybrid zones makes feasible the mapping of loci that contribute to reproductive isolation and trait differences. Heredity.

[b52] Long JC (1991). The genetic structure of admixed populations. Genetics.

[b53] Macholán M, Baird SJE, Dufková P, Munclinger P, Bímová BV, Piálek J (2011). Assessing multilocus introgression patterns: a case study on the mouse x chromosome in central europe. Evolution.

[b54] Mavarez J, Salazar CA, Bermingham E, Salcedo C, Jiggins CD, Linares M (2006). Speciation by hybridization in heliconius butterflies. Nature.

[b55] Mayr E (1942). Systematics and the origin of species from the viewpoint of a zoologist.

[b56] McCullagh P, Nelder J (1989). Generalized linear models.

[b57] Michel AP, Sim S, Powell THQ, Taylor MS, Nosil P, Feder JL (2010). Widespread genomic divergence during sympatric speciation. Proc. Natl Acad. Sci. USA.

[b58] Morjan CL, Rieseberg LH (2004). How species evolve collectively: implications of gene flow and selection for the spread of advantageous alleles. Mol. Ecol.

[b59] Musani SK, Halbert ND, Redden DT, Allison DB, Derr JN (2006). Marker genotypes and population admixture and their association with body weight, height and relative body mass in united states federal bison herds. Genetics.

[b60] Nachman MW, Payseur BA (2012). Recombination rate variation and speciation: theoretical predictions and empirical results from rabbits and mice. Philos. Trans. R. Soc. B.

[b61] Nosil P, Feder JL (2012). Genomic divergence during speciation: causes and consequences. Philos. Trans. R. Soc. B.

[b62] Nosil P, Egan SP, Funk DJ (2008). Heterogeneous genomic differentiation between walking-stick ecotypes: ‘isolation by adaptation’ and multiple roles for divergent selection. Evolution.

[b63] Parra EJ, Marcini A, Akey J, Martinson J, Batzer MA, Cooper R (1998). Estimating african american admixture proportions by use of population-specific alleles. Am. J. Hum. Genet.

[b64] Polechova J, Barton N (2011). Genetic Drift Widens the Expected Cline but Narrows the Expected Cline Width. Genetics.

[b65] Porter AH, Wenger R, Geiger H, Scholl A, Shapiro AM (1997). The *Pontia daplidice-edusa* hybrid zone in northwestern italy. Evolution.

[b66] Pritchard JK, Donnelly P (2001). Case-control studies of association in structured or admixed populations. Theor. Popul. Biol.

[b67] R Development Core Team (2011). R: a language and environment for statistical computing.

[b68] Richards FJ (1959). A flexible growth function for empirical use. J. Exp. Bot.

[b69] Rieseberg LH, Linder CR (1999). Hybrid classification: insights from genetic map-based studies of experimental hybrids. Ecology.

[b70] Rieseberg LH, Raymond O, Rosenthal DM, Lai Z, Livingstone K, Nakazato T (2003). Major ecological transitions in wild sunflowers facilitated by hybridization. Science.

[b71] Rissanen J (1978). Modeling by shortest data description. Automatica.

[b72] Rissanen J, Velupillai KV (2005). Complexity and information in modeling. Computability, complexity and constructivity in economic analysis.

[b73] Slatkin M (1973). Gene flow and selection in a cline. Genetics.

[b74] Szymura JM, Barton NH (1986). Genetic analysis of a hybrid zone between the fire-bellied toads, bombina bombina and b. variegata, near cracow in southern poland. Evolution.

[b75] Turelli M, Orr HA (2000). Dominance, epistasis, and the genetics of postzygotic isolation. Genetics.

[b76] Turner TL, Hahn MW, Nuzhdin SV (2005). Genomic islands of speciation in anopheles gambiae. PLoS Biol.

[b77] Venables WN, Ripley BD (2002). Modern applied statistics with S-Plus.

[b78] Whitham TG (1989). Plant hybrid zones as sinks for pests. Science.

[b79] Wu C-I (2001). The genic view of the process of speciation. J. Evol. Biol.

[b80] Yatabe Y, Kane NC, Scotti-Saintagne C, Rieseberg LH (2007). Rampant gene exchange across a strong reproductive barrier between the annual sunflowers, helianthus annuus and h. petiolaris. Genetics.

